# The network of the subjective experience in embodiment phenomena

**DOI:** 10.1007/s00426-022-01714-7

**Published:** 2022-07-24

**Authors:** Giorgia Tosi, Daniele Romano

**Affiliations:** 1 Department of History, Society and Human Studies, University of Salento, Piazza Tancredi 7, 73100 Lecce, Italy; 2 Department of Psychology, University of Milano - Bicocca, Piazza dell’Ateneo Nuovo 1, 20126 Milan, Italy

## Abstract

**Introduction:**

Body illusions are designed to temporarily alter body representation by embodying fake bodies or part of them. Despite their large use, the embodiment questionnaires have been validated only for the embodiment of fake hands in the rubber hand illusion (RHI).

**Methods:**

With the current study, we aimed at (1) extending the validation of embodiment questionnaires to a different illusory situation e.g., the full-body illusion (FBI); (2) comparing two methods to explore the questionnaires structures: a classic exploratory factor analysis (EFA) and a modern exploratory graph analysis (EGA). 118 healthy participants completed an FBI procedure where the subjective experience of embodiment was measured with a standard questionnaire.

**Results:**

The EFA results in two-factor structures. However, the confirmatory factor analysis (CFA) fit indices do not show a good fit with the data. Conversely, the EGA identified four communities: ownership, agency, co-location and disembodiment; the solution was confirmed by a CFA.

**Conlcusions:**

Overall, the EGA seems to be the best fitting method for the present data. Our results confirm the EGA as a suitable substitute for a more classical EFA. Moreover, the emerged structure suggests that the FBI induces similar effects to the RHI, implying that the embodiment sensations are common to different illusory methods.

## Introduction

In its widest definition, the embodiment hypothesis suggests that human physical, cognitive, and social embodiment ground our conceptual and linguistic systems (Rohrer, 2005). In more philosophical terms, it corresponds to a specific mode of presentation of the property of an object, which results from a specific way the property is processed (de Vignemont, 2011).

We constantly receive different inputs from either the world or the body itself that the brain integrates to create supra-modal mental representations of our own body (Berti, 2013). These representations ensure persistence and coherence to the way we experience our bodies. Nonetheless, they are also constantly changing due to long-term processes such as development (Cowie et al., 2016; Zieber et al., 2010), expertise (Fourkas et al., 2008) and short-term events such as movements (Romano et al., 2019; Wen et al. 2016). Crucially, body representation can be temporarily distorted through experimental procedures, collectively named “body illusions” (Botvinick & Cohen, 1998; Ehrsson et al., 2005; La Roccaet al. 2019; Lenggenhager et al., 2007; Sanchez-Vives et al., 2010; Tosi et al., 2018). In this framework, the term embodiment is used to describe a process where some properties of an external object are processed in the same way as the properties of one’s own body (de Vignemont, 2011).

A particular type of body illusion is the so-called Full-Body Illusion (FBI) in which subjects embody an entire fake body. The FBI can be induced either looking at the fake body from a third- (Lenggenhager et al., 2007; Romano et al., 2014) or a first-person perspective (Banakou et al., 2013; Ehrsson, 2007; Keizer et al., 2016; Petkova & Ehrsson, 2008; van der Hoort et al., 2011). In the context of this paper, we will focus on the FBI from the first-person perspective (simply FBI since now on in this text). In the FBI, participants see a virtual (or filmed) body touched synchronously with their body. The congruence between proprioception, tactile perception and visual feedback has been proved to induce embodiment for the seen body (Ehrsson, 2007; Lenggenhager et al., 2007). The FBI has been replicated using congruent visuotactile stimulations (Romano et al., 2016; van der Hoort et al., 2011) or visuomotor congruency (Kilteni et al., 2012).

Questionnaires are the usual way to measure the experience of embodiment after body illusions; nonetheless, only two studies addressed their psychometric properties (Longo et al., 2008; Romano et al., 2021). Both studies focused on the famous protocol of the rubber hand illusion (RHI), developed by Botvinick and Cohen (1998), that induces the sensation that an external hand belongs to oneself. In the RHI, participants see a fake rubbery hand touched synchronously with their real hand, which is hidden from view. Similarly to the FBI, this illusion reflects a three-way interaction between vision, touch, and proprioception (Botvinick & Cohen, 1998). The anatomical plausibility of the fake hand and the congruency between visual and tactile stimulations induce the embodiment of the rubber hand and the perceptive drift of the real hand towards the fake one. Crucially, the RHI occurs when participants view a compatible rubber hand positioned in a congruent posture that is stimulated synchronously with their hand. On the contrary, the illusion does not work when the tactile stimulation is incongruent with the visual one (asynchronous touch), a no-hand-shaped object is stroked synchronously with the real hand (Tsakiris & Haggard, 2005), or the fake hands are seen in a non-anatomical orientation (Pavani et al., 2000).

Longo and collaborators (2008) designed 27 items (Table 1) based on a qualitative study where participants were asked to describe their experiences during the RHI spontaneously. The items were meant to reflect the sensations participants might have. The authors then asked 130 participants who underwent RHI to indicate their agreement or disagreement with the 27 statements on a 7-item Likert scale, where a response of + 3 indicated that they “strongly agreed” with the statement, a response of − 3 indicated that they “strongly disagreed”, and 0 that they “neither agreed nor disagreed”. The authors performed two independent analyses for the synchronous condition and the asynchronous one. Here, we referred to the synchronous condition to maximizes the overlap with the procedure we wanted to test.

**Table 1:** Full list of items and the factors they were found to load on in the studies by Longo and collaborators (2008) and by Romano et al. (2021)

Item	During the block…	Longo et al., 2008	Romano et al., 2021
1	…it seemed like I was looking directly at my own hand, rather than at a rubber hand	Embodiment (Ownership)	Embodiment
2	…it seemed like the rubber hand began to resemble my real hand	Embodiment (Ownership)	Embodiment
3	…it seemed like the rubber hand belonged to me	Embodiment (Ownership)	Embodiment
4	…it seemed like the rubber hand was my hand	Embodiment (Ownership)	Embodiment
5	…it seemed like the rubber hand was part of my body	Embodiment (Ownership)	Embodiment
6	…it seemed like my hand was in the location where the rubber hand was	Embodiment (Location)	Embodiment
7	…it seemed like the rubber hand was in the location where my hand was	Embodiment (Location)	Embodiment
8	…it seemed like the touch I felt was caused by the paintbrush touching the rubber hand	Embodiment (Location)	Embodiment
9	…it seemed like I could have moved the rubber hand if I had wanted	Embodiment (Agency)	Embodiment
10	…it seemed like I was in control of the rubber hand	Embodiment (Agency)	Embodiment
11	…it seemed like my own hand became rubbery		Embodiment
12	…it seemed like I was unable to move my hand	Loss of own hand	Disembodiment
13	…it seemed like I could have moved my hand if I had wanted	Loss of own hand	Disembodiment
14	…it seemed like I couldn’t really tell where my hand was	Loss of own hand	Disembodiment
15	…it seemed like my hand had disappeared	Loss of own hand	Disembodiment
16	…it seemed like my hand was out of my control	Loss of own hand	Disembodiment
17	…it seemed like my hand was moving towards the rubber hand	Movement	Disembodiment
18	…it seemed like the rubber hand was moving towards my hand	Movement	Disembodiment
19	…it seemed like I had three hands	Movement	
20	I found that experience enjoyable	Affect	
21	I found that experience interesting	Affect	
22	…the touch of the paintbrush on my finger was pleasant	Affect	Physical sensations
23	…I had the sensation of pins and needles in my hand		Physical sensations
24	…I had the sensation that my hand was numb		Physical sensations
25	…it seemed like the experience of my hands was less vivid than normal		
26	…I found myself liking the rubber hand		
27	…it seemed like I was feeling the touch of the paintbrush in the location where I saw the rubber hand being touched		Physical sensations

The principal component analysis (PCA) with a varimax orthogonal rotation led to the extraction of four components which accounted for 55.3% of the variance. The solution was optimized by adopting a varimax orthogonal rotation. The first component comprised items relating to the feelings that the rubber hand was part of the participant’s body (items 1 to 10), and it was termed “embodiment of rubber hand”. The second component, “loss of own hand”, referred to the sensation of losing control of the real hand (items 12 to16). The third component, termed “movement”, was comprised of items relating to the perceived motion of both the real and the fake hand (items 17 to19). The fourth component loaded on items relating to the pleasantness of the experience (items 20 to 22), and the authors named it “affect”. 

The “embodiment of rubber hand” component accounted for 26.3% of the variance. Longo and coworkers, therefore, conducted an additional PCA to inspect any possible sub-components. They identified three components: “ownership” (loading on items related to the feeling that the rubber hand belonged to the participant—items 1 to 5), “location” (related to the feeling that the rubber hand and the real hand were in the same location—items 6 to 8), and “agency” (related to the feeling of control over the fake hand—items 9 and 10).

More recently, Romano and collaborators (2021) furtherly validated the same set of items after inducing the RHI over 298 healthy subjects. The Principal Component Analysis (PCA) on the responses to the synchronous condition suggested a three-components solution explaining 48% of the variance. The first component, named “embodiment”, captured the items about the fake hand embodiment (items 1 to 11). The second component, named “disembodiment”, captured the items related to the loss of control and the fading perception of the real hand (items 12 to18). The authors suggested the term disembodiment by unifying the components of Longo’s solution named “loss of own hand” and “movement”. The third component, named “physical sensations”, captured the items referring to tactile experiences (items 22, 23, 24, 27).

While a few common elements are identifiable in both studies, the entire structure is only partially overlapping, and, more importantly, we still have no proof of how this can be extended beyond the RHI. To the best of our knowledge, validation of any questionnaire for other body illusions is still lacking. In this framework, we aimed to:

(i) Extend the embodiment questionnaire validation to the full-body illusion;
(ii) Compare two methods to explore the questionnaires structures: an Exploratory Factor Analysis (EFA) and an Exploratory Graph Analysis (EGA).

A common procedure to make data reduction is the use of factorial analyses, which individuate a structure of latent variables that are representative of multiple items. Exploratory Factor Analysis (EFA) is typically used in questionnaire data reduction to collapse multiple items to less, more stable and theoretically meaningful dimensions.

EGA is a more recent method that can be used to achieve a similar result from a different perspective. EGA was developed in the context of network models to estimate the number of communities (i.e., latent dimensions) underlying a set of correlated variables (Golino & Epskamp, 2017; Golino et al., 2020a, 2020b). A community is defined as a section of the network where many nodes are connected, and it is considered as resulting from the influence of a latent variable in a network (Golino & Epskamp, 2017). Recently, Golino and coworkers (2020a) investigated the accuracy of EGA in a simulation study by comparing the EGA results with different types of traditional factor-analytical methods. The EGA reached the highest overall accuracy in estimating the number of simulated factors. In a recent preprint, Golino et al. (2020b) address the EGA advantages over more traditional methods: (1) EGA does not demand a rotation method to interpret the estimated factors; (2) EGA automatically distributes items into factors without the researcher’s direction; (3) the network approach shows which community are more central and how items relate within and between communities. We aimed to further compare EGA and EFA on embodiment data.

## Materials and methods

### Participants

The analyses of the current study were run on a composite sample that included data from all the previous experiments run in our laboratory that adopted the Full-Body Illusion with the very same procedure in the synchronous condition (see methods section). The final sample included a total of 118 different healthy volunteers (86 females, mean age 22.42 ± 3.77 years, range 17–48 years; mean education 15.56 ± 1.93 years, range 8–21 years). All participants had normal or corrected to normal vision and were naive to the purpose of the experiment. All the subjects gave their written informed consent before participating in the experiment.

In particular, we included participants from a recent study that validated the procedure with two independent experiments (Tosi et al., 2021). 20 healthy volunteers (15 females; mean age = 22.7 ± 3.0, range 18–27; mean education = 16.1 ± 2.0, range 13–19) and 24 healthy volunteers (13 female; mean age = 25 ± 5.77, range 19–48; mean education = 16.13 ± 1.85, range 13–21), respectively participated in the experiments. Another sample of 41 healthy subjects (38 females, mean age 20.12 ± 2.34 years, range 17–30 years; mean education 13.78 ± 1.46 years, range 12–18 years) took part in another published study about the influence of the full-body illusion on motor affordances (Tosi et al., 2020). The remaining participants are taken from two additional unpublished experiments used for master thesis work at the University of Milano-Bicocca (Experiment 1: 24 participants 14 female; mean age = 22.71 ± 1.90, range 18–26; mean education = 14.71 ± 2.35, range 8–16; Experiment 2: 9 participants, 6 female; mean age = 24.27 ± 1.85, range 21–27; mean education = 17 ± 1, range 16–19).

To determine the adequacy of the sampling size, we referred to the Kaiser–Meyer–Olkin (KMO) test. KMO < 0.5 suggests that the sample size is not adequate for a factor analysis. Our data show an adequate KMO = 0.61. As for the EGA, it is a robust method that showed, under simulation (Golino & Epskamp, 2017), to be less affected by the sample size than other methods for dimension reduction (like EFA). It is safe thus to consider adequate our sample size for both techniques.

The studies were approved by the local Ethics Committees “Commissione per la Valutazione della Ricerca, Dipartimento di Psicologia” of the University of Milano-Bicocca and by the University of Calgary Conjoint Faculties Research Ethics Board (CFREB18-1494). The studies were conducted by the ethical standards of the Declaration of Helsinki (World Medical Association, 2001).

Data and analyses codes are available on the Open Science Framework platform at the following link: https://osf.io/8ba72/?view_only=b433284a9ac54b0a81f6985e72a39482. No part of the study procedures or analysis was pre-registered before the research being conducted.

### Procedure

All participants were exposed to the same Full-Body Illusion procedure, induced through a set of Head-Mounted Displays (HMDs—Samsung Gear VR 2016, Samsung Electronics, field of view = 101° or Oculus Rift, 2018 Oculus VR, field of view = 110°). When we recorded more than one session, we only considered the first one, thus having comparable data across the studies. After the Full-Body Illusion, subjects were asked to rate on a seven-point Likert scale (from − 3 to + 3) their agreement on a 16-statement questionnaire adapted from previous studies evaluating body illusions (Petkova & Ehrsson, 2008; Romano et al., 2016; van der Hoort et al., 2011).

### Body illusion

**Fig. 1:**
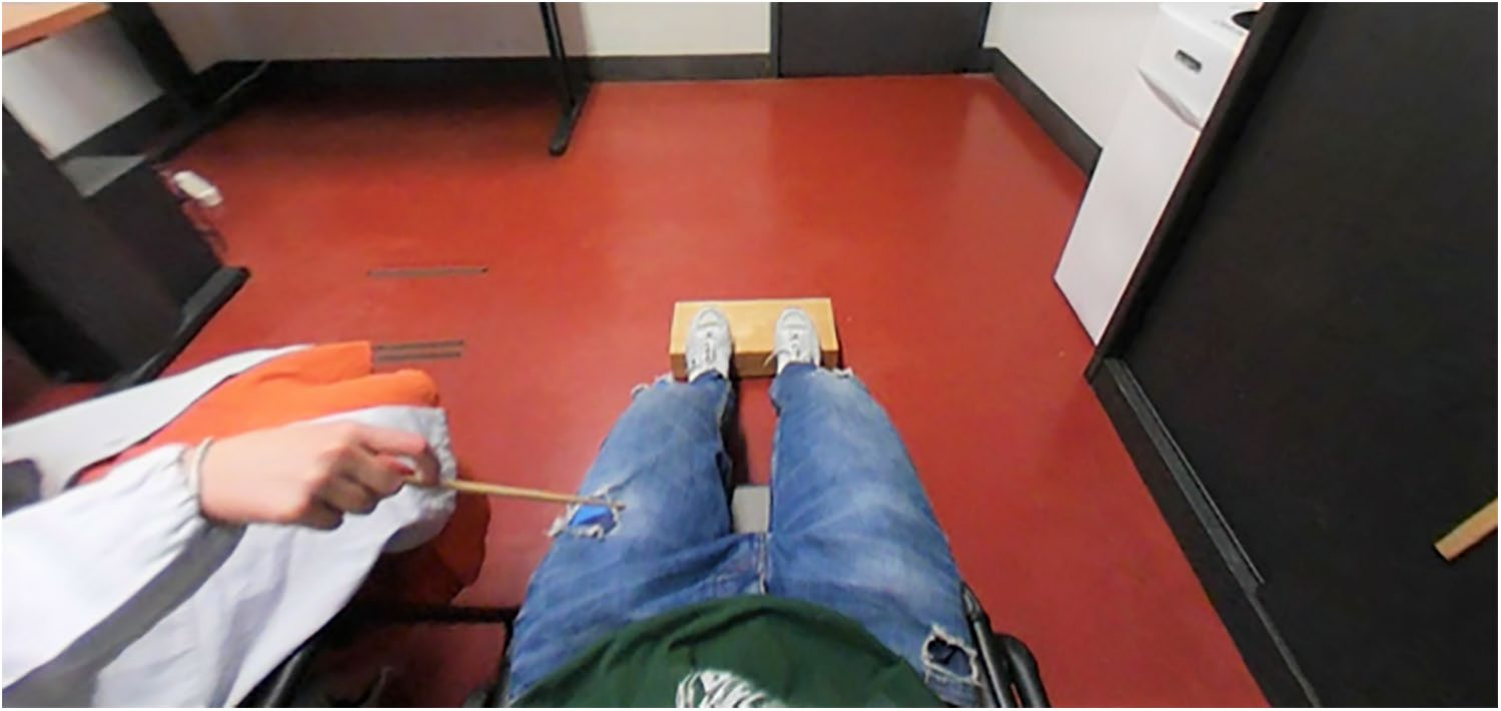
Full-Body Illusion paradigm. The figure shows a frame extracted from the video used to induce the illusion

Participants sat on a chair with their arms lying behind the back of the chair and were invited to look down at their legs and lower abdomen. During the procedure, participants wore a set of head-mounted displays in which they saw a pre-recorded video (encoding: MPEG-H Part2/HEVC (H.265), resolution: 2560 × 1280, 30 fps). The video consisted of a pair of fake legs as seen by a first-person perspective touched by a stick at a frequency of 1 Hz (see Fig. 1). The video was recorded with a 360° camera (Samsung Gear 360 (2016)—camera resolution:15.0 × 2MP; features: CMOS, f/2.0; video recording resolution: near 4k1; processor speed, type: Dual-Core) so that the participants could explore the environment during the video presentation visually. The mannequin presented with averaged legs’ length (108 cm) and upper body size (i.e., it wore a t-shirt of a Medium size). During the video, participants saw the lower abdomen and the legs of the artificial body in an anatomical posture from a first-person perspective; a wooden stick touched the upper left leg for two minutes at a frequency of 1 Hz. Meanwhile, the experimenter delivered a synchronous tactile stimulation touching the participant’s left leg in the corresponding location. Participants heard white noise to avoid any sound interference.

The video is available on the Open Science Framework platform at the following link: https://osf.io/8ba72/?view_only=b433284a9ac54b0a81f6985e72a39482.

### Embodiment questionnaire

At the end of the video, we evaluated the embodiment of the artificial legs with a questionnaire. We adapted 14 statements from the questionnaire proposed by Longo and collaborators (2008) aimed at measuring the experiences induced by the illusion. The original studies of the data also presented in this paper (Tosi et al., 2020, 2021) included two additional items that we dropped from the current analysis because they were addressing aspects specific to those studies (i.e., the perceived size of the fake body and the of the stick). The full list of the items is reported in (Table 2). Participants rated their agreement with the statements on a seven-point Likert scale (from – 3—disagreement – to + 3—agreement). Before the analyses, we reversed items Q4 and Q8.

**Table 2:** Matrix of loadings and variance explained by the components

Item	Did it seem like…	F1 (disembodiment)	F2 (ownership)
Q12	… your own legs became “fake”?	**0.70**	0.18
Q8	… you could have moved your legs if you had wanted?	**0.63**	− 0.09
Q10	… you couldn’t really tell where your legs were?	**0.59**	− 0.25
Q9	… your legs were out of your control?	**0.56**	− 0.15
Q7	… your legs had disappeared?	**0.46**	− 0.10
Q1	… you were looking directly at your own legs?	0.16	**0.65**
Q2	… the legs in the video belonged to you?	0.08	**0.64**
Q3	… you could have moved the legs in the video if you had wanted?	− 0.27	**0.42**
Q4	… you were not in control of the legs in the video?	− 0.17	**0.34**
Q5	… the legs in the video were in the location where your legs were?	− 0.11	0.04
Q6	… the touch you felt was caused by the stick touching the legs in the video?	− 0.16	0.26
Q11	… the experience of your legs was less vivid than normal?	0.22	− 0.01
Q13	Did it seem like you had four legs?	0.13	− 0.23
Q16	Did you find that experience enjoyable?	− -0.22	0.10
*Eigenvalues*		*2.04*	*1.39*
*Proportion Variance*		*0.15*	*0.10*

In bold the loading of each item on the belonging factor. In italics the eigenvalues of each factor and the variance they explain

### Data analysis

The analyses were performed using the *EGAnet* (version 0.9.8; Christensen & Golino 2021), *psych* (Revelle, 2013), and *fa* (Husson, Le, and Pages 2010) packages of the R statistical software (version 4.0.4; R Core Team 2017).

#### Exploratory factor analysis (EFA)

We performed an EFA on the 14 items collected after the Full-Body Illusion. The number of factors was determined considering the eigenvalues > 1, the parallel analysis, the scree-test, and the interpretability of component contents. The solution was Oblimin-rotated to allow for correlation between components.

To further support the solution identified by the FA, we fitted the corresponding model of emergent variables in a Confirmatory Factor Analysis (CFA).

We also inspected potential alternative solutions by adopting a Bass–Ackwards hierarchical procedure (Goldberg, 2006). The Bass–Ackwards procedure explores different levels of specificity among the plausible solutions.

#### Exploratory graph analysis (EGA)

A network is formed by a set of nodes (i.e., the variables of interest) and the edges connecting those nodes (i.e., their relationships). EGA apply the Gaussian Graphical Model (GGM) to estimate the network, followed by dimensions estimation through a communities detection algorithm (i.e., the walktrap algorithm). In GGM, edges represent regularized partial correlation coefficients between two variables after conditioning on all other variables in the dataset (Epskamp & Fried, 2018). To reduce spurious correlation and introduce sparsity, GGM involves the “least absolute shrinkage and selection operator” (LASSO) algorithm (Tibshirani, 1996). The regularization is based on a tuning parameter called lambda (λ), which controls for the sparsity of the network (i.e., the presence of 0-value correlations) (Epskamp, 2016). Lower values of λ remove fewer edges, increasing the possibility of including spurious correlations, larger values of λ remove more edges, increasing the possibility of removing relevant edges. In this study, the ratio of the minimum and maximum λ was set to 0.1. The choice of the best tuning parameter is based on the extended Bayesian Information Criterion (eBIC), which applies a hyperparameter gamma (γ) to control how much it prefers simpler models (i.e., models with fewer edges; Foygel & Drton, 2010). Larger γ values lead to simpler models, while smaller γ values lead to denser models. In the present study, we set γ to 0.25, as suggested in the literature (Epskamp, 2016). Once the network with the smallest eBIC is selected, the EGA uses the walktrap algorithm (Pons & Latapy, 2006) to find the number of communities. The algorithm computes a transition matrix where each element represents the probability of one node traversing to another (based on the sum of the connections to each node). Random walks are then initiated for a certain number of steps (that we set to four) using the transition matrix for probable destinations. Using Ward’s agglomerative clustering approach (Ward, 1963), each node starts as its own cluster and merges with adjacent clusters in a way that minimizes the sum of squared distances between other clusters. Modularity (Newman, 2006) is then used to determine the optimal partition of communities. As a result, a node’s community is determined by its proportion of many densely connected edges to few sparsely connected edges (Christensen & Golino, 2021).

After performing the Exploratory Graph Analysis, we checked for the dimension stability through a bootstrap analysis (Christensen & Golino, 2021). We generated 1000 networks by resampling from the original data with replacement (with the same number of cases as the original data). EGA is then applied to the replicate data, resulting in a sampling distribution of EGA networks. From this sampling distribution, we obtained the median number of dimensions, 95% confidence intervals around the median, and the number of times a certain number of dimensions replicates.

At last, we fitted the corresponding model of latent variables in a Confirmatory Factor Analysis to further support the solution identified by the EGA (Golino et al., 2020a, 2020b).

## Results

### Exploratory factor analysis (EFA)

Two components had eigenvalues > 1. The parallel analysis suggested extracting four components; the scree plot suggested a clear gap after the second component and a smaller gap after the fourth component. Based on these criteria, as well as on an inspection of the content of the items, the best solution was the two components.

This solution explains 25% of the variance, with no correlations between factors. We, therefore, applied a varimax orthogonal rotation to maximize the loadings of the items. We named the first component “disembodiment”, it captures the items relating to the sensation of fading limbs (items Q7, Q8, Q9, Q10, Q12). The disembodiment component shows an internal consistency of α = 0.72. The second component is loaded by items related to the feeling that the fake legs belonged to the participant (items Q1, Q2) and the feelings of control over the fake legs (Q3, Q4); we named it “embodiment”. The embodiment component shows an internal consistency of α = 0.60. Despite most items load on their respective component, a few items (Q5, Q6, Q11, Q13, Q16) show a sub-optimal placement, with a few cross-loadings (see Table 2).

**Fig. 2:**
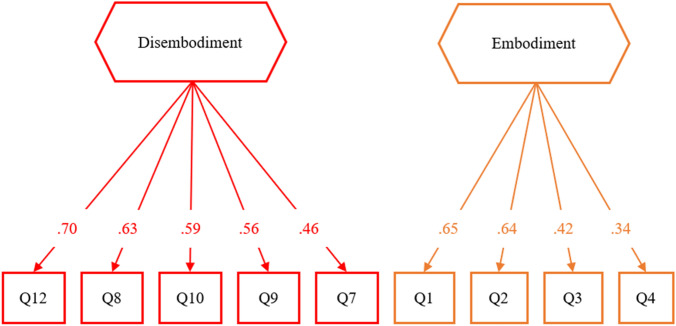
Results of the Exploratory Factor Analysis with a varimax orthogonal rotation. Negative loadings are reported with dash lines, while positive loadings are in solid lines. Colours are used to help the readability of the graph

The two-component solution corresponds to the best model identified by Romano and collaborators (2021) (the third factor of that study included items not adopted here). Figure 2 shows the final model after removing Q5, Q6, Q11, Q13 and Q16. We used the model obtained with the EFA to set the Confirmatory Factor Analysis. The chi-squared (*χ*^2^ (26) = 73.44, *p* < 0.001), the comparative fit index (CFI = 0.74), and the RMSEA = 0.12. indicate a poor fit between the model and the observed data.

We also explored consecutive solutions from a two-component model up to a four-component model, which corresponds to the most complex sustainable solution (justified by the scree plot and the parallel analysis), using a Bass–Ackwards procedure (Goldberg, 2006).

In the three-component solution, the first and the second components keep most of the items of the disembodiment component (Q8, Q9, Q10, Q12) and the embodiment one (Q1, Q2, Q3). The third component retains one item from the previous disembodiment factor (Q7) and one item concerning the location of the fake limb (Q5); we propose to name it “co-location” since it does not recall exactly the location factor proposed by Longo et al. (2008). This solution explains 31% of the variance, with the first and the third factors that are negatively correlated (− 0.29).

The four-component solution keeps the factors disembodiment (Q8, Q9, Q10, Q12) and co-location (Q5, Q7). The embodiment factor splits into the sensation of ownership, with items related to the feeling that the fake legs belonged to the participant (Q1, Q2) and agency, with items related to the feelings of control over the fake legs (Q3, Q4). This solution explains 37% of the variance, with the first and the third factors positively correlated (0.30).

**Fig. 3:**
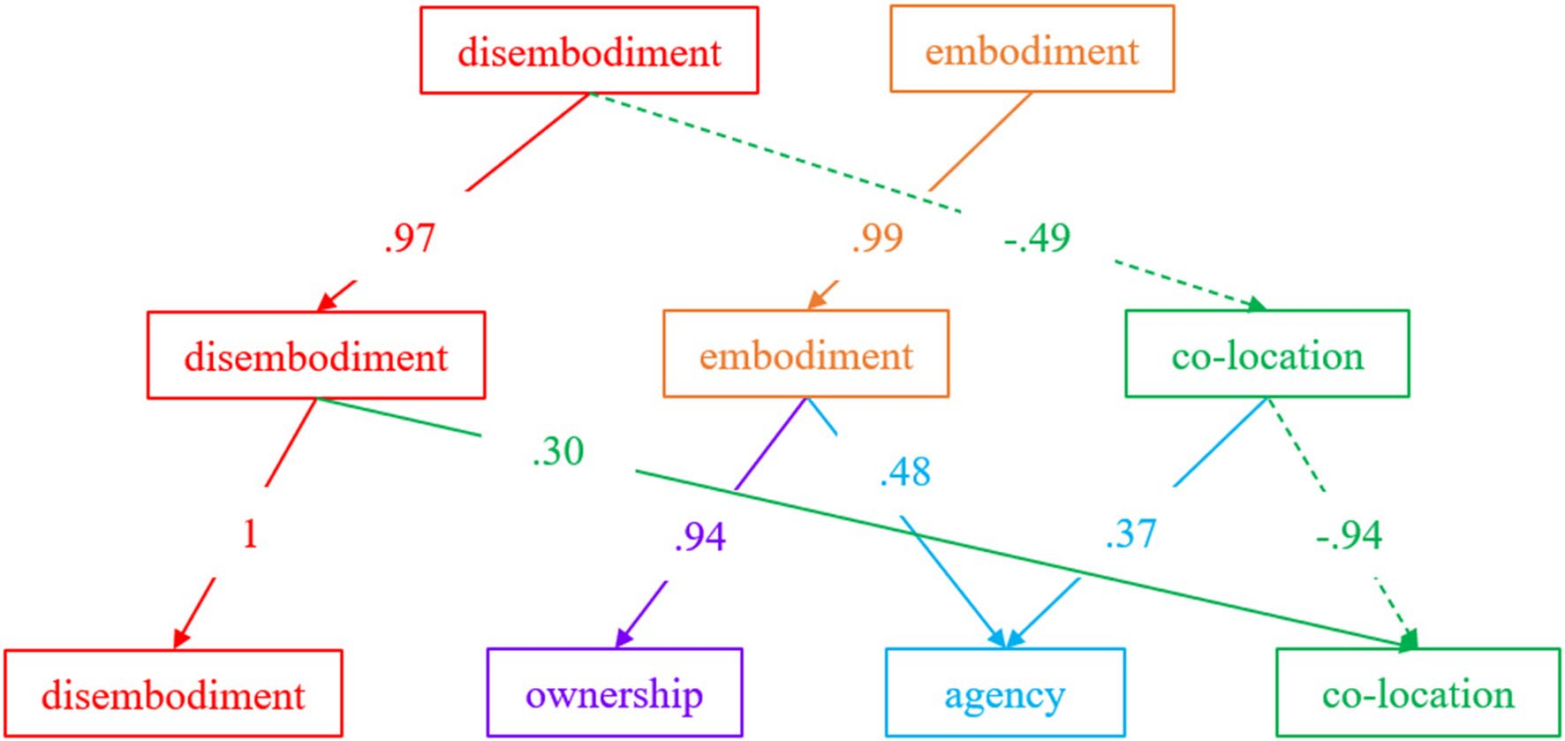
Results of the Bass-Ackwards procedure. The correlations between the components of different solutions are represented. Negative correlations are reported with dash lines, while positive correlations are in solid lines. Colours are used to help the readability of the graph

Correlations between components scores of different level solutions are depicted in (Fig. 3).

The first solution (i.e., the two-factor solution) largely overlap with the structure described by Romano et al. (2021). Across the other solutions, the disembodiment component remains substantially unchanged. The embodiment component splits into two factors (ownership and agency), recovering two out of three subcomponents found by Longo and collaborators (2008). Surprisingly, one item from the disembodiment component (Q7) and one item from the embodiment component (Q5) detach from them and loads on a new component, which we term “co-location”.

#### Exploratory graph analysis (EGA)

**Fig. 4:**
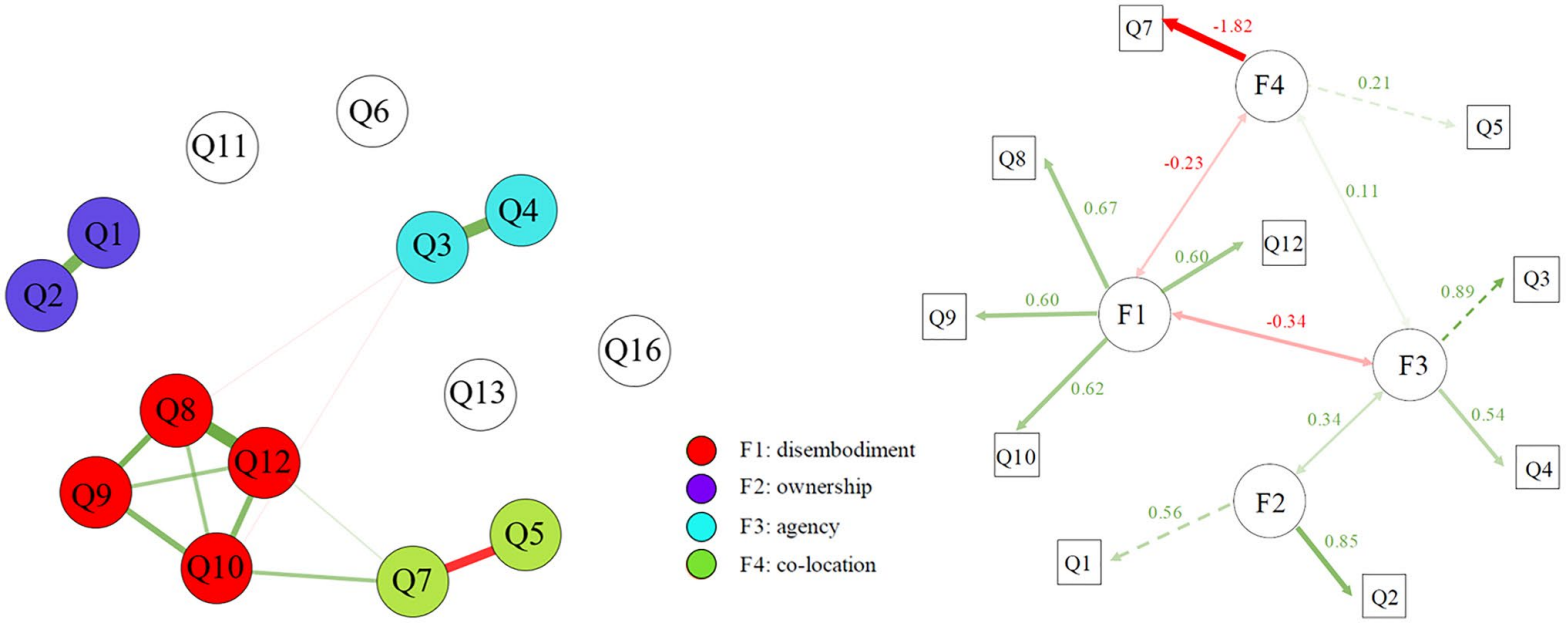
The left panel shows the best network estimated by the Exploratory Graph Analysis. The edges represent regularized partial correlations. Green lines indicate positive associations. Red lines indicate negative associations. The size and the color saturation of the edges represent the intensity of the relationships. The nodes indicate the items in the questionnaire colored by the community they belong to. The right panel shows the Confirmatory Factor Analysis fitted on the EGA results. Items are represented as squares, and latent variables are represented as circles. Green lines indicate positive loadings. Red lines indicate negative loeadings

Figure 4 (left panel) represents the best-estimated network. EGA detected 4 communities, in analogy with one of the sustainable solutions of the EFA and explored with the bass-ackwards procedure. The first community captures the items relating to the sensation of fading limbs (items Q8, Q9, Q10, Q12) similarly to the “disembodiment” factor of the EFA. The second community we termed “ownership” comprehends items relating to the feeling that the fake legs belonged to the participant (items Q1, Q2). The third community reproduces the “agency” factor found in the EFA being formed by items related to the feelings of controlling the fake limbs (Q3, Q4). The fourth community concerns the location of the fake limbs and the disappearance of the real ones (Q5, Q7). Four items (Q6, Q11, Q13, Q16) resulted in being unconnected to the network as their correlations with the other nodes were set to zero in the GGM (see Table 4). Table 3 reports the network loadings. The exact weights of all edges and the simple correlations among all variables are reported in (Table 4)*.*

**Table 3:** Matrix of EGA network loadings

Item	Did it seem like…	F1 (disembodiment)	F1 (ownership)	F3 (agency)	F4 (co-location)
Q5	… the legs in the video were in the location where your legs were?	0.00	**0.23**	0.00	0.00
Q7	… your legs had disappeared?	− 0.06	**0.23**	0.00	0.00
Q8	… you could have moved your legs if you had wanted?	**0.33**	0.00	− 0.03	0.00
Q12	… your own legs became “fake”?	**0.32**	− 0.05	0.00	0.00
Q9	… your legs were out of your control?	**0.26**	0.00	0.00	0.00
Q10	… you couldn’t really tell where your legs were?	**0.23**	− 0.08	− 0.02	0.00
Q3	… you could have moved the legs in the video if you had wanted?	− 0.03	0.00	**0.34**	0.00
Q4	… you were not in control of the legs in the video?	0.00	0.00	**0.34**	0.00
Q1	… you were looking directly at your own legs?	0.00	0.00	0.00	**0.35**
Q2	… the legs in the video belonged to you?	0.00	0.00	0.00	**0.35**

In bold the loading of each item on the belonging community

**Table 4:** The lower part reports simple correlations, measured with Pearson’s. The upper part reports the network weights, which correspond to regularised partial correlations. The diagonal reports the mean and the standard deviation

	Q1	Q2	Q3	Q4	Q5	Q6	Q7	Q8	Q9	Q10	Q11	Q12	Q13	Q16
Q1	0.48 ± 0.81	0.24	0	0	0	0	0	0	0	0	0	0	0	0
Q2	0.48	0.24 ± 0.86	0	0	0	0	0	0	0	0	0	0	0	0
Q3	0.20	0.23	− 0.09 ± 0.89	0.24	0	0	0	− 0.02	0	− 0.01	0	0	0	0
Q4	0.04	0.21	0.48	− 0.15 ± 0.93	0	0	0	0	0	0	0	0	0	0
Q5	− 0.05	− 0.03	0.13	0.12	0.83 ± 0.87	0	− 0.14	0	0	0	0	0	0	0
Q6	0.19	0.14	0.06	0.11	0.00	0.94 ± 0.68	0	0	0	0	0	0	0	0
Q7	0.12	− 0.07	− 0.18	− 0.09	− 0.38	− 0.09	− 0.53 ± 0.94	0	0	0.05	0	0.03	0	0
Q8	0.02	− 0.03	− 0.27	− 0.13	0.05	− 0.12	0.25	− 0.52 ± 0.91	0.15	0.06	0	0.20	0	0
Q9	− 0.12	0.03	− 0.14	− 0.18	0.04	− 0.17	0.23	0.42	− 0.34 ± 0.90	0.10	0	0.07	0	0
Q10	− 0.07	− 0.11	− 0.26	− 0.16	− 0.06	− 0.18	0.30	0.35	0.37	− 0.55 ± 0.79	0	0.12	0	0
Q11	0.05	− 0.11	− 0.06	0.01	0.06	− 0.01	0.01	0.22	0.16	0.08	0.02 ± 0.91	0	0	0
Q12	0.21	0.14	− 0.11	0.03	− 0.03	− 0.10	0.28	0.47	0.36	0.39	0.19	− 0.19 ± 0.95	0	0
Q13	− 0.20	− 0.12	0.03	0.04	− 0.05	− 0.24	0.23	0.01	0.09	0.23	− 0.08	0.06	− 0.93 ± 0.82	0
Q16	0.05	0.06	0.02	0.08	0.01	− 0.07	− 0.19	− 0.12	− 0.16	− 0.24	− 0.13	− 0.06	− 0.10	0.44 ± 0.88

The bootstrap results show that the four dimensions are the most replicable (median = 4, SE = 0.82, 95% CI [2.38; 5.62]). The likelihood of dimensions provides the distribution of the proportion of times that a certain number of dimensions was replicated. This measure further confirms that the four-factors solution was the most frequent (47.6%). The number of factors ranges between one and six, but the other solutions are rarely identified (see Table 5). This analysis confirms that the EGA solution, with the identified four communities, is the most probable dimensional organization.

**Table 5:** The diagonal reports the proportion of times that a certain number of dimensions was replicated during the bootstrap procedure. Each cell reports the proportion between the frequency of the corresponding solutions (column/row)

	F1	F2	F3	F4	F5	F6
F1	0.002	28.500	144.500	238.000	85.000	3.000
F2	0.035	0.057	5.070	8.351	2.982	0.105
F3	0.007	0.197	0.289	1.647	0.588	0.021
F4	0.004	0.120	0.607	0.476	0.357	0.013
F5	0.012	0.335	1.700	2.800	0.170	0.035
F6	0.333	9.500	48.167	79.333	28.333	0.006

The confirmatory factor analysis reported in (Fig. 4) (right panel) validates the four-component solution. The chi-squared (*χ*^2^ (29) = 36.41, *p* = 0.66), comparative fit index (CFI) = 0.94, and the RMSEA = 0.05. indicate a good fit between the model and the observed data.

## Discussion

In the present study, we wanted to investigate the psychometric structure of an embodiment questionnaire in a Full-Body Illusion procedure while comparing two methods to explore latent variables (i.e., EFA and EGA). Previous studies (Longo et al., 2008; Romano et al., 2021) focused on the 27 items designed to capture the embodiment sensation after the Rubber Hand Illusion. Longo and coworkers (2008) described a four-component solution following the synchronous stimulation. The “embodiment” component summarizes all the items relating to the feelings that the rubber hand is part of the participant’s body. This factor splits in three in a second stage analysis, distinguishing between the sense of ownership, the sense of agency, and the sense of co-location of the real and fake hands. The second factor collects items about the sensation of losing the ownership of the real hand (“loss of hand”); the third factor refers to the perceived motion of both the real and the fake hands (“movement”); the fourth factor is related to affective sensations (“affect”). More recently, Romano and coworkers (2021) proposed a simpler three-component solution. The first factor refers to the embodiment of the fake hand and overlaps with the first component by Longo et al. (2008). The second factor gathers the items about the loss of control and the fading perception of the real hand, and it is a sum of the components “loss of own hand” and “movement” proposed by Longo et al. (2008). The authors propose the name “disembodiment”, indicating the decreasing experience of embodiment towards the real hand (della Gatta et al., 2016; Newport et al., 2010, 2011). The last component, “physical sensations”, capture the items referring to tactile experiences.

In our study, we started from fourteen items extracted from the questionnaire proposed by Longo et al. (2008) and adapted to a full-body illusion situation. This set of items has already been used in previous studies (Tosi et al., 2020, 2021), showing that the FBI procedure that we adopted in the present study alters the experience of ones’ body. However, a formal psychometric approach to the questionnaire used to assess the embodiment sensation out of the RHI was lacking.

We performed both a classic EFA and a more modern EGA. The EFA suggests the existence of two components, named “disembodiment” and “embodiment” and confirms the structure found by Romano and coworkers (2021). However, the model does not show a good fit with the data as suggested by the CFA fit indices. On the other hand, the EGA indicates the presence of four components (or communities), and the confirmatory analysis (CFA) shows good fit indices. Our results support the literature (Golino & Epskamp, 2017; Golino et al., 2020a, 2020b) in defining EGA as a more accurate method. Therefore, we are going to discuss the structure proposed by the EGA.

The community “disembodiment” captures the items that Longo (2008) named “loss of own hand” and Romano (2021) “disembodiment”. This community relates to items indicating paralysis of the legs, and the sensation they are turning into fake legs. Romano and collaborators (2021) suggest the embodiment of a fake body part should lead to the real one’s disembodiment. The authors clarify that despite the body representation is keen to include external objects, structural constraints must be respected. Folegatti and co-authors (2012), for example, confirm the impossibility to embody multiple rubber hands. A similar explanation is provided by Longo and coworkers (2008), who imply the fake limb may displace the participant’s actual one. However, looking at the CFA results, the disembodiment community does not correlate with the ownership one. This is the first difference between the FBI and the RHI, where the two components were found to correlate (see Romano et al., 2021).

The second community we found with the EGA is loaded with items related to the feeling that the fake body belongs to the participant and the referral of touch, namely the causal reference between the seen and felt touches (Botvinick & Cohen, 1998). This result is in contrast with the solution proposed by Longo et al. (2008). The authors interpret the causation between the seen and felt touches as evaluating the “location” of the fake limbs. Alternatively, one may consider this item as part of the sense of body ownership: the touch I feel is caused by the stick touching the fake legs because the fake legs belong to me.

Our data suggest the “co-location” component as comprised of items regarding the sense of co-location of the real and fake legs and the proprioception of the real ones. Looking at both the simple correlations and the regularized partial-correlations, the item about perceiving the legs in the video as being in the same location of the real legs (Q5) is negatively correlated with the item about the disappearance of the actual limbs (Q7). In other words, when our participants felt the fake and the real legs being co-located, they did not feel the actual legs disappear, thus perceiving both the real and the fake legs in the same spot at the same moment. Such experience is in contrast with the idea of the disembodiment as opposed to the embodiment; disembodiment does not imply that the fake limb displaces the participant’s actual one.

The fourth community, “agency”, reflects the sensation of motor control over the fake body and concerns the same items as in the previous studies (Longo et al., 2008; Romano et al., 2021).

It is important to note that Longo and coworkers (2008) found the distinction between “ownership”, “agency” and “location” in a second step PCA focusing on the “embodiment” component. In contrast, Romano et al. (2021) suggest a single component solution and recover the subcomponents of the “embodiment” only as a sub-optimal solution. The reason why we were able to distinguish between different components of the main embodiment factor as an optimal solution may stand in the different illusions adopted (RHI vs FBI). When inducing the illusion over a body part, the embodiment experience emerges as a unit and the subcomponents are not recognizable at first sight. On the contrary, if we take into consideration the whole body, as in the FBI, the subcomponents of the embodiment sensation seem to be more independent. A possible limitation of the present study is that the questionnaire items did not refer to the body as a whole. Instead, we specifically focused the questionnaire on the legs because the illusion was part of a broader project regarding the manipulation of legs’ perceived dimensions and metric perception (see Tosi et al., 2020, 2021). Nevertheless, we considered our paradigm as a Full-Body Illusion because participants could see in the video not only their legs but also their chest and lower abdomen, as in real life, we can see our chest, lower abdomen and legs when looking down at our body.

With the FBI, we can elicit the embodiment of a fake body without the concurrent disembodiment of the participant’s actual body. This conclusion is supported by (1) the absence of correlation between the ownership and the disembodiment communities in the CFA solution; (2) the items concerning the location of the fake legs and the disappearance of the actual limbs loading showing a negative correlation. Such disconnection is in line with the original definition of embodiment by de Vignemont (2011): “an object E is embodied if some properties of E are processed in the same way as the properties of one’s body”. The embodiment concerns the processing of an external object as if it is part of our body without replacing it. So, why is the disembodiment sensation so frequent in RHI and not in the FBI?

In a recent work about somatoparaphrenia (SP—i.e., the delusion that one’s limbs belong to someone else), Romano and Maravita (2019) suggest that the defective update of the ongoing dynamic representation of the body may be the key to the disownership feelings of patients with SP. The authors found that the localization of the body affects the feeling of body ownership so that when a body part is located in an unexpected spatial position, it can be attributed to someone else. The failure to update the location of one’s body part in the space may cause its disembodiment as a logical consequence of feeling the body part in a different place (Romano & Maravita, 2019). This work suggests a tight connection between the sense of body ownership and the prediction of where the body is located in space. On this basis, one can argue ownership is not a property of the body but a property of the space where the body is located. If my prediction is that my body is located in a specific spot, a fake body located in the same place will be felt as my body. As a consequence, a tactile stimulus presented in the same location where I predicted my body to be is perceived as touching my body. Accordingly, in our solution, the item about the referral of touch loaded on the ownership community. This result is in line with previous studies reporting a correlation between the referral of touch and the ownership during the RHI (Makin et al., 2008; Reader et al., 2021).

If the sensation of ownership is a matter of space, it can be understood why we did not find a correlation between embodiment and disembodiment. The crucial difference between the RHI and the FBI is indeed the spatial relationship between the fake and the real body or body part. During the RHI the rubber hand is located near the real hand, and several studies found that the proximity of the fake hand to the real limb position plays a key role in the RHI (Lloyd, 2007; Preston, 2013; Preston & Newport, 2011). Following our hypothesis, to embody the fake hand, the participant needs to shift the prediction about where the hand is from the location of the real hand to the location of the rubber one, thus producing the perceptive drift of the real hand towards the rubbery one (Botvinick & Cohen, 1998). The embodiment of the rubber hand demands a shift of the location of the hand, resulting in a lower probability for the original position to host the hand, an effect that can be measured as disembodiment of the real hand (della Gatta, et al., 2016; Newport & Gilpin, 2011; Newport & Preston, 2010). Conversely, during the FBI the real and the fake body are co-located so that the participant does not need to change the prediction about the body location. Both the real and the fake body can coexist because they occupy the same space, where the sensation of ownership is located. Consequently, there is no need to disembody the real body.

Our hypothesis fits with several body illusions and pathological conditions. If we consider the sensation of ownership as a continuum, on the one side SP patients lose ownership over a body part because it is not in the predicted location. On the opposite side, patients affected by the pathological embodiment (PE) condition (Garbarini et al., 2014) likely attribute an alien hand to themselves because it occupies the expected location even in the absence of any other sensory information. Newport and Gilpin (2011) were able to induce the somatoparaphrenic sensation of disownership over a body part in healthy subjects. In the disappearing hand trick, the authors made the participants’ right hand disappear from view using a sensorimotor adaptation procedure, in which the hands slowly, and without the subjects’ awareness, moves outwards. Thus, when the participants were asked to reach the perceived location of the right hand, it was not there anymore. The participants failed to update the location of the right hand and consequently reported the sensation that it was no longer part of their body. Conversely, patients suffering from Phantom Limb feel ownership over a body part after its amputation. In line with our hypothesis, patients predict the limb to be where it used to be and allocate the sensation of ownership. First described in the treatment of phantom limb pain (Ramachandran et al., 1995), the Mirror Box (MB) induces embodiment over the reflection of a healthy limb. The reflection of the healthy hand seems visually superimposed on the felt location of the phantom, creating the illusion that the phantom has been resurrected (Ramachandran & Altschuler, 2009). Romano and collaborators (2013) suggested that the critical trigger of the MB is the “visual capture” effect (Botvinick & Cohen, 1998; Holmes et al., 2004; Pavani et al., 2000) where the visual input is weighted more than the signals coming from the hidden hand (Van Beers et al., 1998). We can interpret the visual capture as a consequence of the location prediction. The visual stimulus is weighted more than the proprioceptive signals because it is located where I predict the phantom to be.

Future studies may directly address the relationship between the sense of body ownership and disownership, manipulating the co-location and the perspective of the real and fake body.

Our second aim was to compare two explorative approaches to data reduction: the EGA based on network analysis and the classic EFA. When Golino and Epskamp (2017) proposed the exploratory graph analysis, they compared it to traditional techniques to estimate the number of dimensions underlying simulated data. In their work, EGA performed comparably to parallel analysis and Kaiser-Guttman eigenvalue > 1 rule. In a more recent simulation study, Golino and coworkers (2020) compared the EGA with traditional factor-analytical methods. The EGA showed the highest estimation accuracy. In the present paper, we propose EGA as an alternative method to identify the embodiment questionnaire structure. This method suggested the extraction of four communities, confirmed by a CFA that showed excellent fit indices. On the contrary, the EFA found a simpler solution with two components and a poor fit with the data. The four-factor solution was retrieved only with a Bass-Ackward procedure assessing a more complex solution.

The EGA seems to be the best fitting method for the present data; additionally, it gives an item-level look into the correlations between the items. Our results confirm the EGA as a suitable substitute for a more classical exploratory factor analysis. As an advantage, EGA automatically identifies which items indicate the retrieved dimensions. To the best of our knowledge, the number of dimensions to be extracted cannot be pre-set since EGA is an exploratory method. In light of this point, EGA may be considered less flexible than EFA. However, EGA allows checking for the dimensional stability through bootstrap analysis.

The non-parametric bootstrap procedure that we used generates data by resampling with replacement from the original dataset, allowing us to estimate the dimensions stability and their replication rate (Christensen & Golino, 2021). Crucially, the selection of the number of dimensions can be based on the frequency of each solution replicate during the bootstrapping. If two solutions have a similar replication rate, they are roughly equally probable, and one can consider examining both structures.

Another plus of the EGA is the possibility to check the solution by fitting the corresponding Confirmatory Factor Analysis (CFA) (Golino et al., 2020a, 2020b). CFA is a confirmatory technique driven by theoretical relationships among observed and unobserved variables (Schreiber et al., 2006). By assuming the EGA result as a theoretical model, the CFA returns different goodness of fit indices allowing to confirm the network structure. Our results indicate a good fit between the model and the observed data, supporting the potential of using network analysis to estimate the number of latent dimensions underlying a set of variables.

## Conclusion

Despite several differences between the FBI and the RHI, our study suggests that the core of the embodiment experience remains the same across different body illusions. Nonetheless, a few specificities have been specified for the two illusions. These differences gave insight into the possible cognitive mechanisms that underlie the sense of ownership, highlighting the importance of the different illusions in the panorama of the cognitive sciences.
